# A Case–Control Study of Factor V Leiden G1691A and MTHFR A1298C Polymorphisms and Clinical Outcomes in Patients With COVID‐19

**DOI:** 10.1002/iid3.70493

**Published:** 2026-07-27

**Authors:** Hilal Çıralıoğlu, Yasemin Adalı, Mert Özen, Alten Oskay, Atakan Yılmaz, Murat Seyit, Aylin Köseler, İbrahim Türkçüer

**Affiliations:** ^1^ Department of Emergency Medicine Pamukkale University Faculty of Medicine Denizli Türkiye; ^2^ Centre for Public Health, School of Medicine, Dentistry and Biomedical Sciences Queen's University Belfast Belfast UK; ^3^ Division of Epidemiology, Department of Public Health, Faculty of Medicine Pamukkale University Denizli Türkiye; ^4^ Department of Biophysics Pamukkale University Faculty of Medicine Denizli Türkiye

**Keywords:** case–control study, COVID‐19, d‐dimer, epidemiology, Factor V Leiden, mortality, MTHFR, thrombophilia

## Abstract

**Objective:**

The clinical relevance of inherited thrombophilia‐related polymorphisms in COVID‐19 remains uncertain. This study investigated the distribution of Factor V Leiden (FVL) G1691A and MTHFR A1298C polymorphisms in patients with COVID‐19 and associations with laboratory findings and short‐term mortality.

**Methods:**

This case‐control study included 150 patients with PCR‐confirmed COVID‐19 and 300 controls. Genomic DNA was isolated from peripheral blood, and FVL G1691A and MTHFR A1298C polymorphisms were analyzed. Associations with d‐dimer levels, hospitalization duration, and 30‐ and 90‐day mortality were evaluated using multivariable linear and Cox regression analyses.

**Results:**

The FVL GG genotype was less frequent in patients than controls (77.3% vs. 87.0%, *p* = 0.009), whereas the MTHFR AA genotype was more frequent (76.7% vs. 61.3%, *p* = 0.001). Heterozygosity in both genes did not differ between groups (8.0% vs. 6.3%, *p* = 0.552). Patients with heterozygosity in both genes had higher d‐dimer levels (median 435.0 vs. 201.0 ng/mL, *p* = 0.024) and longer hospitalization (median 7.5 vs. 5.0 days, *p* = 0.037). After adjustment, heterozygosity in both genes remained associated with higher log‐transformed d‐dimer levels (*β* = 0.758, 95% CI: 0.239–1.277; *p* = 0.005), as did MTHFR A1298C (*β* = 0.432, 95% CI: 0.007–0.856; *p* = 0.046). None of the polymorphisms was associated with 30‐ or 90‐day mortality, while age independently predicted both outcomes.

**Conclusion:**

Thrombophilia‐related polymorphisms may influence d‐dimer levels and hospitalization duration in COVID‐19 but do not appear to affect short‐term mortality.

## 1 Introduction

Viral pneumonia is a severe disease capable of causing global pandemics with high morbidity and mortality. Coronavirus disease 2019 (COVID‐19) has been associated with significant endothelial injury, microvascular abnormalities, an exaggerated inflammatory response, and increased procoagulant activity [1]. A systematic review and meta‐analysis also demonstrated the thrombotic and microangiopathic effects of severe acute respiratory syndrome coronavirus 2 (SARS‐CoV‐2), as well as the incidence and risk of thromboembolism in patients with COVID‐19 [2]. However, whereas some patients with COVID‐19 develop marked hypercoagulability and thromboembolic complications, others recover without thromboembolic events [3]. This variation suggests that individual susceptibility factors, including hereditary thrombophilia, may contribute to clinical outcomes.

Factor V and Factor VIII play important roles in the coagulation cascade by promoting activation of Factor X and accelerating the conversion of prothrombin to thrombin [4]. Protein C, Protein S, and antithrombin act as natural anticoagulants by limiting excessive clot formation [4]. Factor V Leiden (FVL) is the most common inherited thrombophilia and is characterized by resistance to activated Protein C. The clinical risk associated with FVL‐related thrombophilia depends on the number of mutant alleles. In individuals with the heterozygous genotype, the risk of thrombosis is increased by approximately 3‐ to 8‐fold, whereas in homozygous individuals, this risk may increase by 18‐ to 80‐fold [5]. The heterozygous FVL genotype has been reported in approximately 3%–8% of the general population in the United States and Europe [6].

Methylenetetrahydrofolate reductase (MTHFR) is an important enzyme in folate metabolism and homocysteine regulation [7]. Polymorphisms in the MTHFR gene may reduce enzyme activity and contribute to elevated plasma homocysteine levels, which have been associated with endothelial dysfunction and thrombotic risk [7, 8]. Elevated homocysteine levels have also been linked to atherosclerosis and cardiovascular disorders, including coronary artery disease, myocardial infarction, and ischemic stroke [8]. In this context, MTHFR polymorphisms have attracted attention as possible contributors to thrombotic susceptibility.

Given the prothrombotic nature of COVID‐19 and the potential contribution of inherited thrombophilic variants, investigating these genetic polymorphisms may help to better understand interindividual differences in disease severity and prognosis. Therefore, this study aimed to evaluate the distribution of Factor V Leiden G1691A and MTHFR A1298C gene polymorphisms in patients with COVID‐19 and to investigate their associations with clinical outcomes and mortality.

## 2 Materials and Methods

### 2.1 Study Population

This single‐center descriptive‐analytical case‐control study was conducted at the Department of Emergency Medicine, Pamukkale University Faculty of Medicine Hospital, between June 10, 2021 and April 10, 2023. Ethical approval was obtained from the Pamukkale University Non‐Invasive Clinical Research Ethics Committee (decision dated June 10, 2021; approval no. 60661).

Patients presenting to the emergency department with symptoms such as cough, sputum, fever, and shortness of breath were evaluated according to the Turkish Ministry of Health COVID‐19 Diagnosis and Treatment Guideline. Patients with PCR‐confirmed COVID‐19 were included in the patient group. This group comprised 150 patients, including both mild cases without radiological evidence of pneumonia on lung computed tomography and patients who required ward or intensive care unit admission according to their clinical condition.

The control group consisted of 300 healthy volunteers without active respiratory symptoms, known chronic disease, regular medication use, or a history of COVID‐19 or any other infection within the previous 6 months.

### 2.2 Inclusion and Exclusion Criteria

The inclusion criteria for the patient group were age ≥ 18 years and PCR‐confirmed COVID‐19 infection. Patients were evaluated clinically and radiologically, and both mild cases without radiological evidence of pneumonia and patients requiring hospitalization were included. The exclusion criteria were chronic kidney disease, chronic liver disease, known inflammatory disease, major metabolic or endocrine disorders including non‐euthyroid thyroid disease, chronic obstructive pulmonary disease, asthma, known heart failure, referral from another center while intubated, and a history of cancer or active malignancy.

### 2.3 Data Collection

Age, sex, symptom duration, vital signs, comorbidity status, laboratory findings (biochemistry, hemogram, and coagulation parameters), radiological findings, and gene polymorphism results were recorded. In addition, clinical disposition (discharge, ward admission, or intensive care unit admission), hospitalization duration, CURB‐65 and PSI scores, and mortality outcomes were documented.

### 2.4 Genetic Analysis

Approximately 2 mL of peripheral blood was collected from each participant into anticoagulant‐containing (K3EDTA) vacuum tubes. Genomic DNA was isolated using the standard phenol‐chloroform method. Relevant gene‐specific regions were amplified by polymerase chain reaction (PCR), and polymorphic loci were evaluated on high‐resolution agarose gel. Genotyping was then confirmed based on DNA sequence analysis.

Primers for MTHFR A1298C polymorphism

Forward: 5′‐CAAGGAGGAGCTGCTGAAGA‐3′

Reverse: 5′‐CAACTCCAGCATCACT‐3′

Primers for Factor V Leiden G1691A polymorphism

Forward: 5′‐CATACTACAGTGACGTGGAC‐3′

Reverse: 5′‐TGTTCTCTTGAAGGAAATGC‐3′

### 2.5 Statistical Analysis

Descriptive statistics were presented as mean ± standard deviation (SD), median with interquartile range (IQR), minimum‐maximum, and frequency (%), as appropriate. The distribution of continuous variables was assessed using the Shapiro–Wilk test together with distributional characteristics. For parametric comparisons, homogeneity of variance was also evaluated. Welch's *t* test was used for approximately normally distributed continuous variables when appropriate, whereas the Mann–Whitney *U* test was used for non‐normally distributed variables. Categorical variables were compared using Pearson's chi‐square test or Fisher's exact test, as appropriate. In addition to *p* values, effect size measures were calculated to better reflect the magnitude of observed differences. Effect size was presented as *r* for Mann‐Whitney *U* tests, Hedges’ *g* for parametric comparisons, phi coefficient (*φ*) for 2 × 2 categorical comparisons, and Cramér's V for categorical variables with more than two categories.

To evaluate short‐term mortality, Cox proportional hazards regression analyses were performed for 30‐day and 90‐day mortality. Unadjusted and adjusted hazard ratios (HRs) with 95% confidence intervals (95% CIs) were calculated. Age, sex, and comorbidity were included in the adjusted models a priori based on their established clinical relevance in COVID‐19 prognosis and their potential role as confounders in the association between thrombophilia‐related polymorphisms and short‐term mortality. Because of the limited number of mortality events, each polymorphism variable was evaluated in a separate model to reduce the risk of overfitting. The proportional hazards assumption was assessed for each Cox regression model using Schoenfeld residual‐based tests and was found to be acceptable.

Because d‐dimer values showed a right‐skewed distribution, log‐transformed d‐dimer was used as the dependent variable in linear regression analyses. Unadjusted and adjusted linear regression models were used to examine the association between genetic polymorphisms and log‐transformed d‐dimer levels. Adjusted linear regression models included age, sex, and comorbidity, selected a priori based on clinical relevance and confounding potential. Beta coefficients (*β*) and 95% confidence intervals (95% CIs) were reported. All statistical analyses were performed using Stata IC version 16.1.

## 3 Results

### 3.1 Baseline Characteristics of the Study Population

The baseline characteristics of the patient and control groups are presented in Table 1. The mean age was 53.4 ± 20.7 years in the patient group and 52.6 ± 21.1 years in the control group, with no significant difference between groups (*p* = 0.628). Among the patients, 63 (42.0%) were female and 87 (58.0%) were male. In the patient group, 83 individuals (55.3%) had at least one comorbid disease.

**Table 1:** Baseline Characteristics of the Patient and Control Groups.

Variable	Patient group (*n* = 150) *n* (%)/mean ± SD	Control group (*n* = 300) *n* (%)/mean ± SD	Effect size	*p* value
Age, years	53.4 ± 20.7	52.6 ± 21.1	*r* = 0.023	0.628^a^
Sex			*φ* = 0.079	0.095^b^
Female	63 (42.0)	151 (50.3)		
Male	87 (58.0)	149 (49.7)		
FVL G1691A			*φ* = 0.124	0.009^b^
GG	116 (77.3)	261 (87.0)		
GA	34 (22.7)	39 (13.0)		
MTHFR A1298C			*φ* = 0.153	0.001^b^
AA	115 (76.7)	184 (61.3)		
AC	35 (23.3)	116 (38.7)		
Heterozygosity for FVL G1691A and MTHFR A1298C			*φ* = 0.031	0.552^b^
No	138 (92.0)	281 (93.7)		
Yes	12 (8.0)	19 (6.3)		

*Note:* Effect size is presented as r for the Mann‐Whitney *U* test and phi coefficient (φ) for categorical comparisons.

a Mann‐Whitney *U* test,

b Pearson's chi‐square test

Regarding genotype distribution, the FVL G1691A GG genotype was observed in 116 patients (77.3%) and the GA genotype in 34 patients (22.7%). For MTHFR A1298C, 115 patients (76.7%) had the AA genotype and 35 (23.3%) had the AC genotype. Heterozygosity in both genes was identified in 12 patients (8.0%).

No significant differences were found between the patient and control groups in terms of age or sex distribution. However, significant differences were observed in genotype frequencies. The GG genotype of FVL G1691A was more common in the control group than in the patient group (87.0% vs. 77.3%, *p* = 0.009), whereas the AA genotype of MTHFR A1298C was more frequent in the patient group than in the control group (76.7% vs. 61.3%, *p* = 0.001). No significant difference was found between the groups regarding heterozygosity in both genes (*p* = 0.552) (Table 1).

### 3.2 Laboratory Findings According to Heterozygosity Status

**Figure 1:**
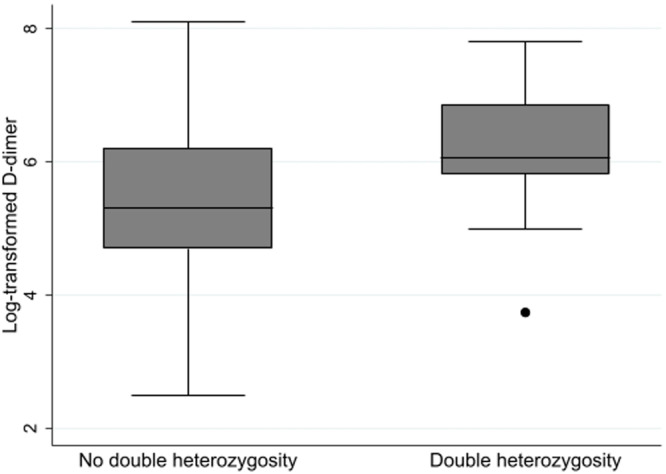
Distribution of log‐transformed d‐dimer levels according to double heterozygosity status for FVL G1691A and MTHFR A1298C polymorphisms.

Laboratory findings according to heterozygosity in both genes are presented in Table 2. Among the evaluated laboratory parameters, only d‐dimer levels differed significantly between groups. Patients with heterozygosity in both genes had higher d‐dimer levels than those without double heterozygosity (median 435.0 [IQR, 613.5] vs. 201.0 [IQR, 387.0] ng/mL, *p* = 0.024). The distribution of log‐transformed d‐dimer levels according to double heterozygosity status is shown in Figure 1.

**Table 2:** Comparison of Laboratory Findings According to Double Heterozygosity for FVL G1691A and MTHFR A1298C.

Parameter	No double heterozygosity (*n* = 138)	Double heterozygosity (*n* = 12)	Effect size	*p* value
WBC (×10^3^/µL)	7.85 (IQR: 5.57)	8.58 (IQR: 4.87)	*r* = 0.014	0.865^a^
Hemoglobin (g/dL)	13.2 ± 2.3	12.7 ± 2.4	*g* = −0.206	0.509^c^
Neutrophils (×10^3^/µL)	5.13 (IQR: 4.29)	6.20 (IQR: 4.59)	*r* = 0.025	0.761^a^
Lymphocytes (×10^3^/µL)	1.49 (IQR: 1.37)	1.63 (IQR: 1.31)	*r* = 0.008	0.922^a^
Platelets (×10^3^/µL)	235.3 ± 81.0	243.6 ± 95.2	*g* = 0.100	0.775^c^
Monocytes (×10^3^/µL)	0.79 (IQR: 4.43)	0.67 (IQR: 1.76)	*r* = 0.057	0.483^a^
CRP (mg/L)	20.58 (IQR: 82.98)	36.62 (IQR: 86.76)	*r* = 0.042	0.608^a^
Urea (mg/dL)	30.0 (IQR: 21.0)	26.5 (IQR: 36.0)	*r* = 0.013	0.873^a^
Creatinine (mg/dL)	0.87 (IQR: 0.37)	0.84 (IQR: 0.59)	*r* = 0.059	0.469^a^
d‐dimer (ng/mL)	201.0 (IQR: 387.0)	435.0 (IQR: 613.5)	*r* = 0.185	0.024^a^
Ferritin (ng/mL)	145.5 (IQR: 379.14)	108.5 (IQR: 269.2)	*r* = 0.051	0.528^a^
Troponin	6.11 (IQR: 16.88)	7.77 (IQR: 12.49)	*r* = 0.004	0.961^a^
Fibrinogen (mg/dL)	318.5 (IQR: 211.0)	403.5 (IQR: 251.0)	*r* = 0.082	0.317^a^
INR (ratio)	1.09 (IQR: 0.19)	1.06 (IQR: 0.10)	*r* = 0.077	0.348^a^

*Note:* Continuous variables are presented as median (IQR) for non‐normally distributed variables and mean ± SD for approximately normally distributed variables. Effect size is presented as r for Mann–Whitney *U* test and Hedges’ g for parametric comparisons.

a Mann‐Whitney U test.

c Welch's *t* test.

No statistically significant differences were observed between the groups for WBC, hemoglobin, neutrophil count, lymphocyte count, platelet count, monocyte count, CRP, urea, creatinine, ferritin, troponin, fibrinogen, or INR (all *p* > 0.05) (Table 2).

### 3.3 Clinical and Radiological Findings According to Heterozygosity Status

Clinical and radiological findings are summarized in Table 3. No significant differences were observed between patients with and without heterozygosity in both genes in terms of body temperature, SpO_2_, presence of COVID‐19‐related symptoms at admission, symptom duration, radiological findings, CT severity, clinical status, hospitalization unit among hospitalized patients, CURB‐65 score, or PSI score (all *p* > 0.05).

**Table 3:** Clinical and radiological characteristics according to double heterozygosity for FVL G1691A and MTHFR A1298C polymorphisms.

Variable	No double heterozygosity (*n* = 138)	Double heterozygosity (*n* = 12)	Effect size	*p* value
Body temperature	36.65 (IQR: 0.40)	36.85 (IQR: 0.55)	*r* = 0.094	0.250^a^
SpO_2_	95 (IQR: 5)	95 (IQR: 3)	*r *= 0.039	0.635^a^
Symptom status			*φ* = 0.066	0.342^b^
Absent	13 (9.4)	2 (16.7)		
Present	125 (90.6)	10 (83.3)		
Symptom duration			*V* = 0.134	0.260^c^
Asymptomatic	7 (5.1)	2 (16.7)		
1–10 days	114 (82.6)	9 (75.0)		
≥ 10 days	17 (12.3)	1 (8.3)		
Radiological finding			*φ* = 0.098	0.259^b^
Normal	26 (18.8)	4 (33.3)		
Pneumonia	112 (81.2)	8 (66.7)		
CT severity			*V* = 0.034	0.917^c^
Normal	42 (30.4)	3 (25.0)		
Mild–moderate	66 (47.8)	6 (50.0)		
Severe	30 (21.7)	3 (25.0)		
Clinical status			*φ* = 0.074	0.511^b^
Discharged	40 (29.0)	2 (16.7)		
Hospitalized	98 (71.0)	10 (83.3)		
Hospitalization unit among hospitalized patients			*φ* = 0.037	0.708^b^
Ward	74 (75.5)	7 (70.0)		
Intensive care unit	24 (24.5)	3 (30.0)		
Hospitalization duration	5.0 (IQR: 7.0)	7.5 (IQR: 5.5)	*r* = 0.171	0.037^a^
CURB‐65 score	1.0 (IQR: 2.0)	1.5 (IQR: 1.5)	*r* = 0.110	0.178^a^
PSI score	68.5 (IQR: 76.0)	80.0 (IQR: 87.5)	*r* = 0.002	0.983^a^

*Note:* Continuous variables are presented as median (IQR). Effect size is presented as *r* for Mann–Whitney *U* test, phi coefficient (*φ*) for 2 × 2 categorical comparisons, and Cramér's V for categorical variables with more than two categories.

a Mann‐Whitney U test.

b Fisher's exact test.

c Pearson's chi‐square test.

However, hospitalization duration was significantly longer in patients with heterozygosity in both genes than in those without heterozygosity (median 7.5 [IQR, 5.5] vs. 5.0 [IQR, 7.0] days, *p* = 0.037) (Table 3).

### 3.4 Cox Regression Analyses for 30‐Day and 90‐Day Mortality

Cox proportional hazards regression analyses for 30‐day and 90‐day mortality are presented in Table 4. In unadjusted analyses, neither FVL G1691A, MTHFR A1298C, nor heterozygosity in both genes was significantly associated with 30‐day or 90‐day mortality. Similarly, after adjustment for age, sex, and comorbidity, none of these polymorphisms showed a significant association with either 30‐day or 90‐day mortality.

**Table 4:** Cox proportional hazards regression analyses for 30‐day and 90‐day mortality.

Variable	30‐day HR (95% CI)	*p* value	90‐day HR (95% CI)	*p* value
FVL G1691A (GA vs GG)				
Unadjusted	1.57 (0.55–4.53)	0.401	1.45 (0.51–4.10)	0.489
Adjusted	1.79 (0.60–5.36)	0.297	1.58 (0.54–4.65)	0.405
MTHFR A1298C (AC vs AA)				
Unadjusted	1.08 (0.35–3.36)	0.889	1.00 (0.33–3.07)	0.999
Adjusted	1.52 (0.48–4.77)	0.473	1.41 (0.45–4.36)	0.554
Heterozygosity in both genes (+ vs −)				
Unadjusted	1.68 (0.38–7.41)	0.491	1.58 (0.36–6.89)	0.546
Adjusted	2.36 (0.51–10.88)	0.270	2.16 (0.47–9.84)	0.321
Age (per year)				
Adjusted	1.06 (1.02–1.10)	0.004	1.06 (1.02–1.10)	0.004

*Note:* Adjusted models included age, sex, and comorbidity, selected a priori based on clinical relevance and confounding potential.

Each polymorphism variable was evaluated in a separate model because of the limited number of mortality events.

During follow‐up, 17 deaths occurred within 30 days and 18 deaths occurred within 90 days.

The proportional hazards assumption was assessed using Schoenfeld residual‐based tests and was found to be acceptable for all models.

**Figure 2:**
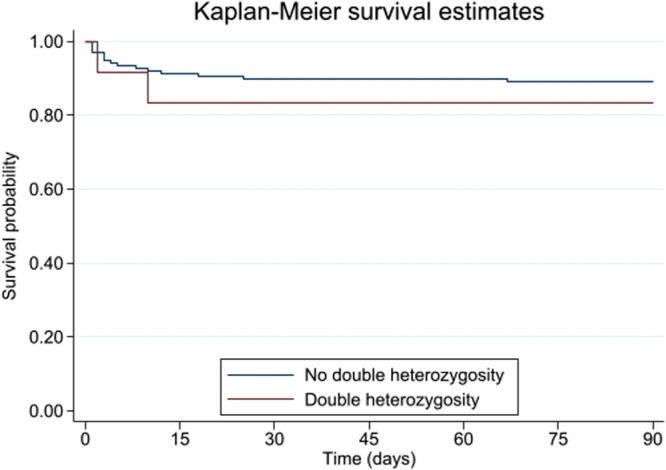
Kaplan–Meier curves for 90‐day survival according to double heterozygosity status for FVL G1691A and MTHFR A1298C polymorphisms.

For 30‐day mortality, the adjusted hazard ratios were 1.79 (95% CI: 0.60–5.36; *p* = 0.297) for FVL G1691A, 1.52 (95% CI: 0.48–4.77; *p* = 0.473) for MTHFR A1298C, and 2.36 (95% CI: 0.51–10.88; *p* = 0.270) for heterozygosity in both genes. Comparable findings were obtained for 90‐day mortality. The adjusted hazard ratios were 1.58 (95% CI: 0.54–4.65; *p* = 0.405) for FVL G1691A, 1.41 (95% CI: 0.45–4.36; *p* = 0.554) for MTHFR A1298C, and 2.16 (95% CI: 0.47–9.84; *p* = 0.321) for heterozygosity in both genes. In contrast, age was independently associated with both 30‐day and 90‐day mortality in the adjusted analyses (HR: 1.06, 95% CI: 1.02–1.10, *p* = 0.004 for both models) (Table 4). Kaplan‐Meier curves for 90‐day survival according to double heterozygosity status are shown in Figure 2.

### 3.5 Linear Regression Analyses for d‐Dimer

Unadjusted and adjusted linear regression analyses for log‐transformed d‐dimer levels are shown in Table 5. In unadjusted analyses, FVL G1691A was not associated with log‐transformed d‐dimer levels (*β* = 0.108, 95% CI: −0.318 to 0.533; *p* = 0.618). Similarly, MTHFR A1298C was not significant in the unadjusted model (*β* = 0.261, 95% CI: −0.211 to 0.733; *p* = 0.276). However, heterozygosity in both genes was significantly associated with higher log‐transformed d‐dimer levels in the unadjusted model (*β* = 0.740, 95% CI: 0.110 to 1.371; *p* = 0.022).

**Table 5:** Unadjusted and adjusted linear regression analyses for log‐transformed d‐dimer levels.

Variable	Unadjusted *β* (95% CI)	*p* value	Adjusted *β* (95% CI)	*p* value
FVL G1691A (GA vs GG)	0.108 (−0.318 to 0.533)	0.618	0.052 (−0.316 to 0.419)	0.781
MTHFR A1298C (AC vs AA)	0.261 (−0.211 to 0.733)	0.276	0.432 (0.007 to 0.856)	0.046
Heterozygosity in both genes (+ vs −)	0.740 (0.110 to 1.371)	0.022	0.758 (0.239 to 1.277)	0.005

*Note:* Log‐transformed d‐dimer was used as the dependent variable because of the skewed distribution of raw d‐dimer values.

Adjusted models included age, sex, and comorbidity, selected a priori based on clinical relevance and confounding potential.

After adjustment for age, sex, and comorbidity, heterozygosity in both genes remained significantly associated with higher log‐transformed d‐dimer levels (*β* = 0.758, 95% CI: 0.239 to 1.277; *p* = 0.005). In addition, MTHFR A1298C was significantly associated with higher log‐transformed d‐dimer levels in the adjusted model (*β* = 0.432, 95% CI: 0.007 to 0.856; *p* = 0.046), whereas FVL G1691A remained non‐significant (*β* = 0.052, 95% CI: −0.316 to 0.419; *p* = 0.781) (Table 5).

## 4 Discussion

In the present study, genotype distributions of FVL G1691A and MTHFR A1298C differed between patients with COVID‐19 and healthy controls. Among patients with COVID‐19, double heterozygosity for both polymorphisms was associated with higher d‐dimer levels and longer hospitalization duration, whereas neither individual polymorphism nor double heterozygosity was independently associated with 30‐day or 90‐day mortality. These findings suggest that thrombophilia‐related polymorphisms may be more relevant to coagulation‐related laboratory abnormalities and selected intermediate clinical outcomes than to short‐term survival in this cohort.

One of the main findings of this study was the association between heterozygosity in both genes and elevated d‐dimer levels. COVID‐19 is well known to be associated with coagulation abnormalities, thrombotic complications, and abnormal hemostatic markers, particularly in hospitalized patients and severe cases [1, 3, 9, 10]. In our cohort, patients with heterozygosity in both genes had significantly higher d‐dimer levels than those without double heterozygosity, and this association remained significant after adjustment for age, sex, and comorbidity. In addition, MTHFR A1298C showed a borderline association with increased log‐transformed d‐dimer levels after adjustment. Given the exploratory nature of these analyses and the relatively small number of patients with double heterozygosity, these findings should be interpreted cautiously. Nevertheless, they raise the possibility that inherited thrombophilia‐related variants may contribute to coagulation‐related biomarker variation in COVID‐19.

The biological plausibility of these findings is supported by the known roles of FVL and MTHFR in thrombophilia. FVL is one of the most prominent inherited thrombophilia‐related variants and is associated with activated protein C resistance and an increased risk of venous thromboembolism [4, 5]. Similarly, inherited thrombophilia has been discussed as a potential modifier of thrombotic risk in COVID‐19, especially in the setting of endothelial injury, inflammation, and hypercoagulability [1, 11, 12]. MTHFR polymorphisms, although less consistently linked to thrombosis than FVL, have also been associated with folate metabolism, methylation pathways, endothelial dysfunction, and inflammatory responses [12, 13, 14]. In this context, our findings suggest that the combined presence of these polymorphisms may be more relevant to coagulation activation, reflected by d‐dimer elevation, than to mortality.

Another important finding was the longer hospitalization duration observed in patients with double heterozygosity for FVL G1691A and MTHFR A1298C. Patients with double heterozygosity had a longer median hospital stay than those without double heterozygosity, which may indicate a more prolonged clinical course in this subgroup [15, 16]. However, no significant differences were observed in most other clinical and radiological variables, including symptom status, symptom duration, radiological findings, CT severity, CURB‐65 score, PSI score, or hospitalization unit among hospitalized patients. Therefore, although these polymorphisms may be associated with selected intermediate outcomes such as d‐dimer levels and hospitalization duration, they do not appear to be consistently associated with overall short‐term clinical severity across the measured domains.

Importantly, none of the investigated polymorphisms was significantly associated with 30‐day or 90‐day mortality in either unadjusted or adjusted Cox regression analyses. This is a clinically relevant finding, as it suggests that FVL G1691A and MTHFR A1298C should not be interpreted as independent predictors of short‐term mortality in patients with COVID‐19. Previous studies in this area have reported mixed findings. Some authors have suggested that thrombophilia‐related genetic background may influence clinical severity or thrombotic burden in COVID‐19 [10, 17, 18, 19], whereas others have emphasized the dominant role of acquired inflammatory and coagulation disturbances over inherited predisposition [1, 2, 11]. Our findings are more consistent with the latter interpretation and suggest that these polymorphisms may influence biomarkers such as d‐dimer without necessarily translating into increased short‐term mortality.

The absence of an independent mortality association should be interpreted together with the strong and consistent effect of age in our study. Age remained independently associated with both 30‐day and 90‐day mortality, which is fully consistent with the established literature on COVID‐19 outcomes. In this setting, traditional clinical risk factors may remain more important determinants of prognosis than thrombophilia‐related polymorphisms [20]. Likewise, although more than half of our patients had comorbidities, comorbidity status was not independently associated with mortality in the adjusted models [21, 22]. This may reflect limited statistical power, the broad categorization of comorbidity, or the heterogeneity of underlying conditions.

Our findings also add to the limited literature examining combined genetic susceptibility. Although FVL and MTHFR have individually been discussed in relation to thrombosis and COVID‐19 severity, fewer studies have focused on the coexistence of thrombophilia‐related polymorphisms in the same patient [16, 19, 23]. In our study, heterozygosity in both genes was associated with higher d‐dimer levels and longer hospitalization, suggesting that combined genetic predisposition may be more informative for coagulation‐related and intermediate outcomes than single‐variant analyses alone. However, given the limited number of patients with heterozygosity in both genes, these findings should be interpreted cautiously and require external validation.

## 5 Limitations

This study has several limitations. It was conducted at a single center and included a relatively small sample, which may limit generalizability. No homozygous carriers of FVL G1691A or MTHFR A1298C were identified, restricting genotype‐specific analyses. In addition, the use of healthy volunteers as controls may have introduced selection bias. Comorbidities were not analyzed in detail by subtype, and residual confounding cannot be excluded despite adjusted analyses. Finally, the limited number of short‐term mortality events may have reduced the power to detect modest associations in survival analyses.

## Conclusion

In conclusion, in this case–control study, double heterozygosity for FVL G1691A and MTHFR A1298C was associated with higher d‐dimer levels and longer hospitalization duration among patients with COVID‐19, and MTHFR A1298C also showed a modest association with higher log‐transformed d‐dimer levels after adjustment. However, neither the individual polymorphisms nor double heterozygosity was independently associated with 30‐day or 90‐day mortality. Overall, these findings suggest that thrombophilia‐related polymorphisms may be linked to selected coagulation‐related and intermediate clinical outcomes rather than short‐term survival, although confirmation in larger multicenter studies is needed.

## Author Contributions

Laboratory and Medical Practices: Hilal Çıralıoğlu, Aylin Köseler, İbrahim Türkçüer. Concept: Hilal Çıralıoğlu, Aylin Köseler, İbrahim Türkçüer. Design: Hilal Çıralıoğlu, Aylin Köseler, İbrahim Türkçüer. Data Collection or Processing: Hilal Çıralıoğlu, Alten Oskay, Atakan Yılmaz, Mert Özen, Murat Seyit, İbrahim Türkçüer. Analysis or Interpretation: Hilal Çıralıoğlu, Yasemin Adalı, Aylin Köseler, Mert Özen, İbrahim Türkçüer. Literature Search: Hilal Çıralıoğlu, Yasemin Adalı, Alten Oskay, Atakan Yılmaz, Mert Özen, Murat Seyit, İbrahim Türkçüer. Writing: Hilal Çıralıoğlu, Yasemin Adalı, Aylin Köseler, İbrahim Türkçüer.

## Data Availability

The data used in this study are available from the corresponding author upon reasonable request.
